# Navigating Cytomegalovirus Retinitis in a Patient With Myelodysplastic Syndromes Transitioning to Acute Myeloid Leukemia Post Transplant: A Case Study

**DOI:** 10.7759/cureus.78491

**Published:** 2025-02-04

**Authors:** Steven A Benyahia, Sunny Kahlon, Srijit Paul, Yusuf Aboul-Nasr, Kevin Harvey, Mamta Patel, Shivan Shah, Enas Abdallah

**Affiliations:** 1 Internal Medicine, University of South Florida Morsani College of Medicine, Tampa, USA; 2 Ophthalmology, University of South Florida Morsani College of Medicine, Tampa, USA; 3 Infectious Diseases, Moffitt Cancer Center, Tampa, USA; 4 Malignant Hematology, Moffitt Cancer Center, Tampa, USA

**Keywords:** acute myeloid leukemia (aml), cmv retinitis, graft versus host disease, myelodysplastic syndromes (mds), ul97 resistance

## Abstract

We present a case of cytomegalovirus (CMV) retinitis with UL97 resistance in a patient undergoing treatment for acute myeloid leukemia (AML), highlighting the complex interplay between hematological malignancies and CMV management. A 49-year-old female patient with myelodysplastic syndromes (MDS) with 4.5% blasts and TP53 mutations ((variant allele frequency (VAF)) 5.2%) underwent an allogeneic stem cell transplant complicated by acute graft versus host disease (GvHD) and subsequent CMV viremia. Her UL97 resistance posed significant challenges to CMV management, necessitating a transition from ganciclovir to foscarnet and maribavir. The CMV polymerase chain reaction (PCR) levels initially >10,000 copies/mL decreased to <300 copies/mL with combination therapies, though fluctuations persisted until letermovir prophylaxis was initiated. Six months after the transplant, AML relapse was treated with cladribine, cytarabine, granulocyte-colony-stimulating factor/filgrastim, and mitoxantrone (CLAG-M) and venetoclax, leading to measurable clearance of TP53 mutations to undetectable levels by NGS and reduction of CD34+ blasts from 25% to 0%. However, the patient developed vision loss and was diagnosed with CMV retinitis, requiring intravitreal and systemic foscarnet alongside laser prophylaxis for retinal detachment. The complexity of her case was compounded by foscarnet-induced acute kidney injury (AKI), requiring intravenous hydration and suspected GvHD, managed with steroids and supportive care. This case illustrates the aggressive nature of MDS-AML progression, the critical role of vigilant CMV surveillance, and the nuanced approach needed for treatment, balancing efficacy against potential side effects. The successful navigation of these complexities offers valuable insights for similar cases.

## Introduction

Cytomegalovirus (CMV) is a herpesvirus that can cause severe and life-threatening complications. Cytomegalovirus retinitis can present as a serious ocular complication, particularly in immunocompromised patients, including those undergoing cancer treatment. This condition is known for its potential severity in individuals with diminished immune responses and can progress to vision loss. While the incidence of CMV retinitis has decreased with improved treatment of HIV/AIDS, its prevalence has increased among patients undergoing chemotherapy or on immunomodulatory medications [1]. Despite being commonly associated with HIV patients, CMV retinitis is rare in hematopoietic stem cell transplantation (HSCT) recipients unless they are significantly immunosuppressed, often due to complications like graft versus host disease (GvHD) [2]. One retrospective study of 4,241 HSCT recipients found that 25.06% developed CMV viremia, and 1.58% progressed to CMV retinitis, highlighting its relatively rare occurrence in this population despite significant immunosuppression [3]. This complexity in identifying its root causes, strongly linked to various immunodeficiency disorders, underscores its continued significance in the fields of infectious diseases and ophthalmology [4]. The landscape of CMV retinitis has been reshaped with the advent of antiretroviral therapy (ART) [5]. The symptoms of CMV retinitis are diverse, ranging from reduced visual acuity to complete visual loss. Complications can result, such as retinal detachment and immune recovery uveitis [6, 7].

Acute myeloid leukemia (AML) and myelodysplastic syndromes (MDS) are hematological malignancies that compromise the immune system through bone marrow failure and ineffective blood cell production. Myelodysplastic syndromes can progress to AML, with both conditions increasing susceptibility to opportunistic infections like CMV due to prolonged immunosuppression [8]. Hematopoietic stem cell transplantation, a treatment used to replace damaged bone marrow in these patients, further heightens the risk of CMV reactivation due to pre-transplant conditioning regimens, ongoing immunosuppressive therapies, and post-transplant complications like GvHD, a condition where donor immune cells attack the recipient’s tissues [9].

This case report presents a 49-year-old woman diagnosed with CMV retinitis in the right eye, with a particular focus on her unique clinical presentation and immunological profile. Her case is especially pertinent as she was undergoing treatment for AML, highlighting the complex interplay between cancer, particularly hematological malignancies like AML, and the risk of developing CMV retinitis.

## Case presentation

A 49-year-old female with a past medical history of gastroesophageal reflux disease (GERD), migraines, kidney stones, and hyperlipidemia presented with fatigue, dyspnea, and intermittent fever. A CBC ordered by her primary care physician revealed pancytopenia, prompting a referral to an oncologist. A subsequent bone marrow biopsy demonstrated findings consistent with MDS, leading to her presentation at Moffitt Cancer Center, Tampa, FL, for further evaluation.

**Figure 1:**
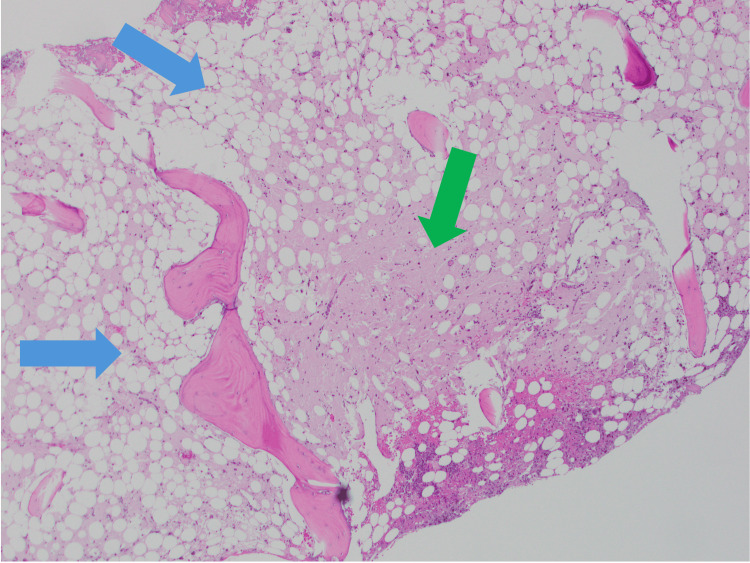
Bone marrow biopsy core (x4 magnification) Low-power view of the bone marrow biopsy demonstrating hypoplastic marrow with significant fat infiltration and areas of multilineage dysplasia. Note the lighter staining areas indicative of hypocellularity with a fat component (blue arrows) and marrow damage as seen in the pink acellular area (green arrow).

**Figure 2:**
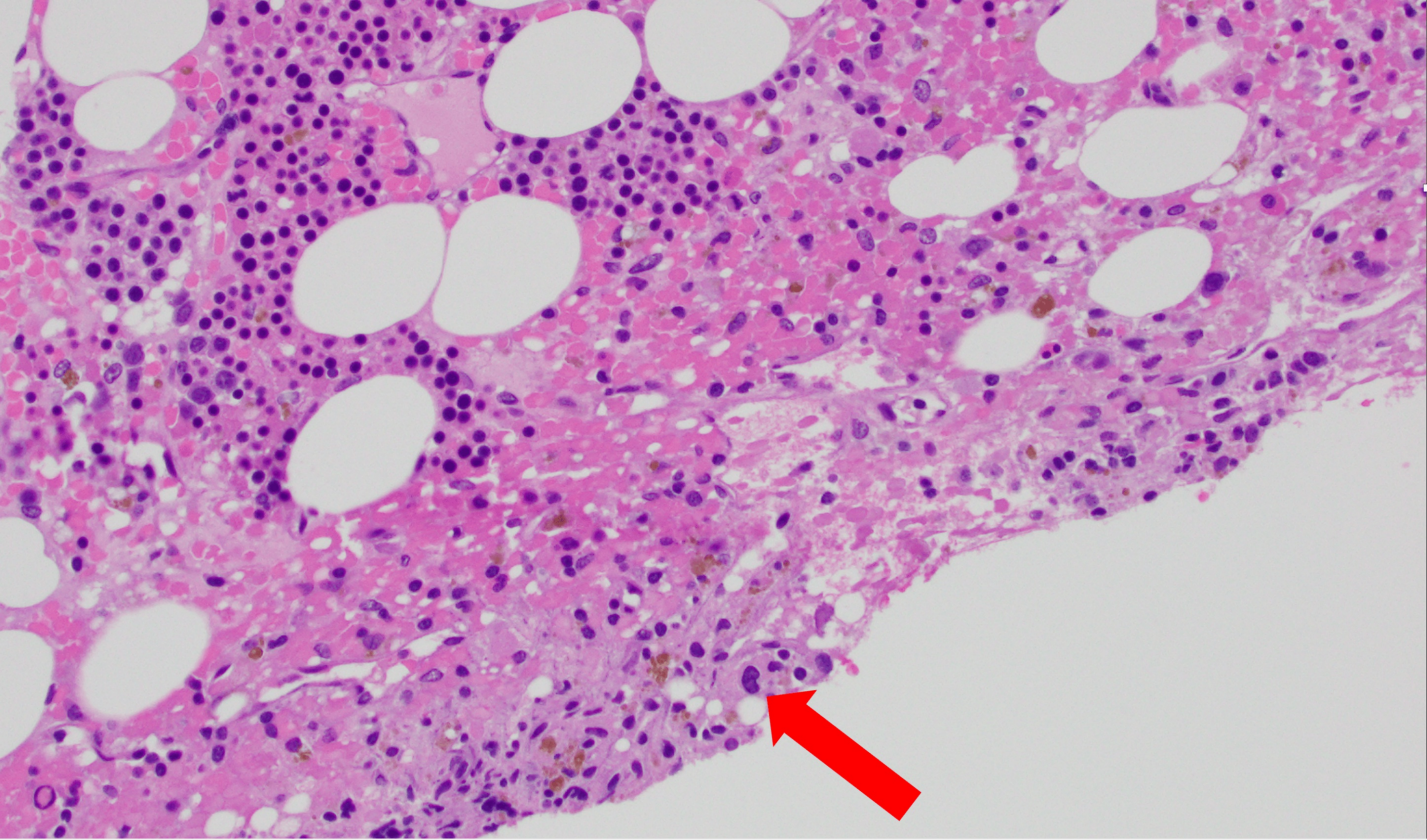
Bone marrow biopsy core (x20 magnification) High-power view highlighting dysplastic changes in hematopoietic cells, consistent with myelodysplastic syndrome (MDS) with multilineage dysplasia (MLD). The only dysplastic features that can be seen are occasional megakaryocytes with small hypolobated nuclei (indicated by the red arrow), which contrast with the normal cells' more uniform appearance.

One month later (May 2022), a repeated bone marrow analysis confirmed MDS with multilineage dysplasia (MLD) with 4.5% blasts via CD34 stain (Figures 1-2). The MDS fluorescence in situ hybridization (FISH) panel was negative, but the myeloid next-generation sequencing (NGS) panel revealed TP53 mutations. Her International Prognostic Scoring System-Revised (IPSS-R) score was > six, consistent with high-risk MDS. After discussing treatment options, she was enrolled in a clinical trial, receiving azacitidine plus magrolimab/placebo. After two cycles of treatment, a follow-up bone marrow analysis showed 0% blasts, no mitotic activity on cytogenetics, and negative MDS FISH but a persistent TP53 mutation. She completed three treatment cycles of the clinical trial, followed by an allogeneic hematopoietic stem cell transplantation (allo-HSCT) with a matched unrelated donor (MUD) in September of 2022, which was complicated with acute GvHD of the GI tract treated with tacrolimus and prednisone. Her lab work in November 2022 showed a positive CMV polymerase chain reaction (PCR) in the blood, consistent with CMV viremia.

Initial treatment included IV ganciclovir, the standard of care for CMV, followed by oral valganciclovir as maintenance therapy; however, the patient demonstrated an inadequate response, with persistent CMV viremia, suggesting possible antiviral resistance. The patient received foscarnet induction therapy for three days due to concerns about ganciclovir resistance. Despite temporary improvement (positive CMV but levels <300), her CMV levels fluctuated, necessitating alternating treatments between valganciclovir 900 mg twice a day (BID), foscarnet (IV and intra-vitreal), and eventually settled on maribavir (an oral antiviral with activity against UL97-mutated CMV) as salvage therapy. By April 2023, the CMV levels remained persistent, and the patient continued maribavir.

Unfortunately, six months post transplant (March 2023), a bone marrow biopsy showed dysplastic changes with increased blasts (~8% by CD34 immunostaining). TP53 immunostaining was strongly expressed in more than 10% of cells. Chimerism confirmed relapse (89% donor) consistent with relapsed disease. She underwent one cycle of oral decitabine and cedazuridine (Inqovi), followed by hospitalization for severe pneumonia with respiratory failure and poor count recovery, halting further cycles. With some count recovery and intolerance to Inqovi, she completed two cycles of IV decitabine, but a subsequent biopsy showed hypocellular marrow (10% to 15%) with dyspoiesis and increased blasts (20% to 25% by CD34 stain), consistent with AML with mutated TP53. In June 2023, she was admitted for cladribine, cytarabine, granulocyte-colony-stimulating factor/filgrastim, and mitoxantrone (CLAG-M) + venetoclax treatment. A follow-up biopsy showed no evidence of AML and NGS clearance of TP53 mutations.

**Figure 3:**
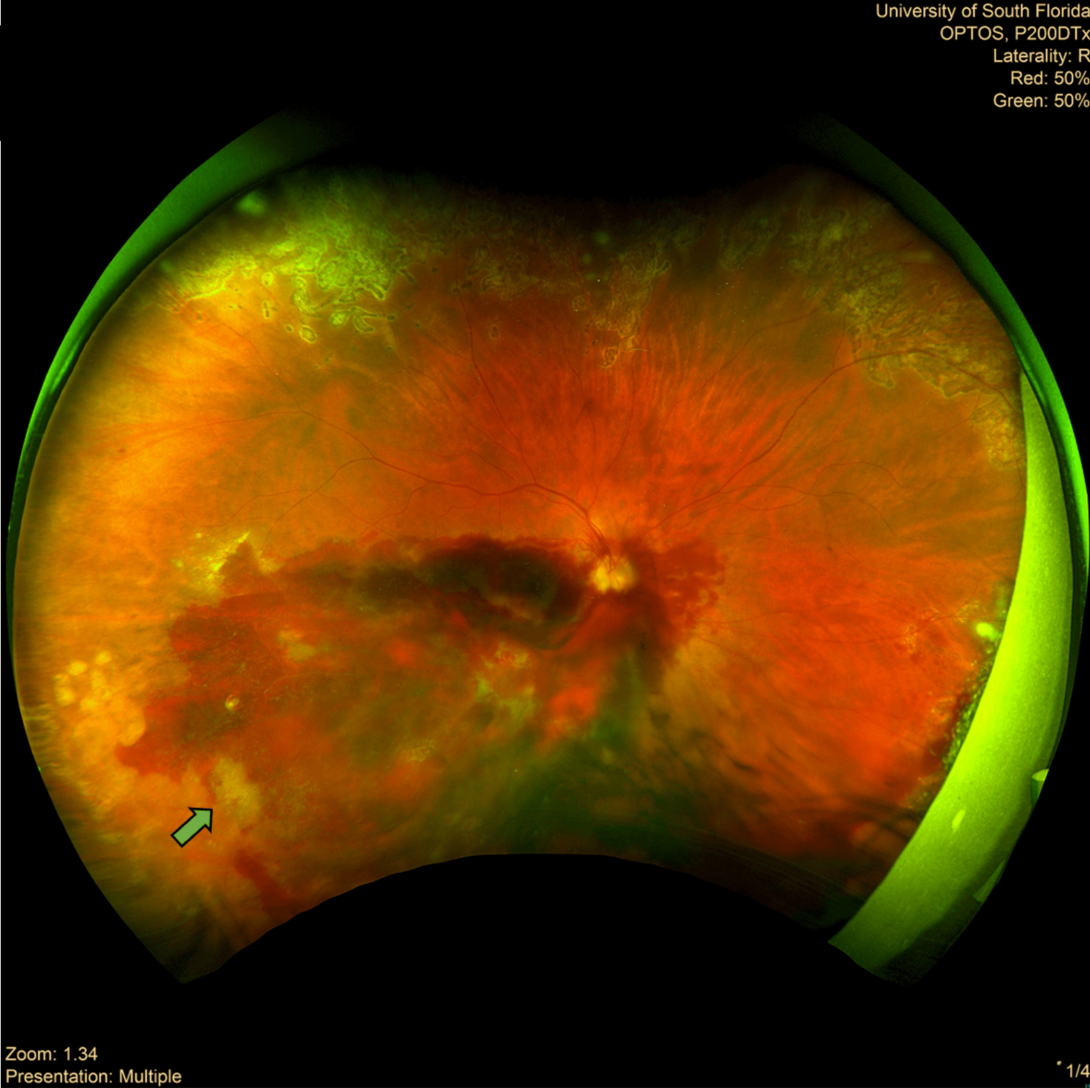
Right eye Optos wide-field retinal image Obtained two weeks after prophylactic pan-retinal photocoagulation (PRP) of the eye was performed. Areas of chorioretinal scarring from PRP can also be seen toward the periphery (green arrow). This is in contrast to a healthy retina that would display a uniform reddish-orange hue with an intact vascular network, smooth pigmentation, and a well-defined macula and optic disc.

**Figure 4:**
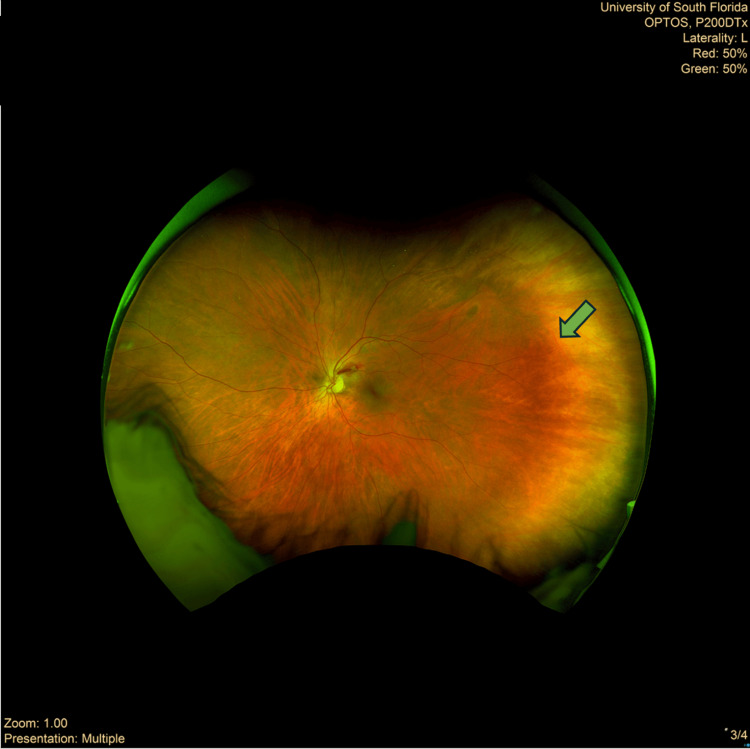
Left eye Optos wide-field retinal image The flame-shaped hemorrhage (green arrow) with possible adjacent microaneurysm indicates bleeding within the nerve fiber layer of the retina, a common finding in patients with severe thrombocytopenia.

However, in early August 2023, she developed vision loss in her right eye. The CT scan of the head and an ultrasound of the eye were negative for hemorrhage. She saw her ophthalmologist, who had a concern for CMV retinitis. She was found to have significant UL97 resistance with codon H520Q with significant ganciclovir resistance. She was treated with both IV and intravitreal injections of foscarnet in her right eye. Infectious diseases and ophthalmology departments coordinated care. She received foscarnet and maribivir for CMV retinitis and underwent weekly intravitreal injections. During her treatment, she was administered pan-retinal photocoagulation laser therapy as prophylaxis against retinal detachment (Figure 3). Examination of the patient’s left eye revealed a flame-shaped hemorrhage with a possible adjacent microaneurysm (Figure 4). It was thought to be secondary to the patient’s thrombocytopenia (platelet count: 41,000/μL; reference range: 150,000-450,000/uL). Subsequently, she underwent vitrectomy surgery for a non-clearing vitreous hemorrhage.

As a consequence, the platelet threshold was increased to >50,000/μL. She experienced an acute kidney injury (AKI) likely due to foscarnet, which improved with hydration and the addition of pre- and post-foscarnet boluses. She was discharged home after completing four weeks of intravenous foscarnet BID in the bone marrow transplant (BMT) treatment center.

A follow-up revealed a worsening eye condition with bleeding. Further intravitreal foscarnet was not recommended, but an increase in platelet threshold to >50,000/μL was suggested. She also experienced uncontrolled nausea, vomiting, and diarrhea and was found to have possible Grade 1 GvHD with negative CMV testing. She is currently on prednisone, budesonide, and beclomethasone, showing improvement in symptoms. As of the latest update, her condition has been stable, with a decreased platelet threshold of > 30,000/μL.

## Discussion

This case report of a 49-year-old female patient with MDS transitioning to AML and subsequently developing CMV retinitis post transplant presents several unique aspects worth discussing. The uniqueness of this case lies in the complex interplay of MDS, post-transplant complications, and the rare occurrence of CMV retinitis in this patient demographic, which is not commonly reported in the literature.

The progression of MDS to AML is a well-documented phenomenon, occurring in approximately 30% of MDS patients [8]. Additionally, the progression of MDS with TP53 mutation to AML is documented to occur in approximately 41% of patients [10]. However, the rapid progression in this case, despite aggressive treatment including a clinical trial with azacitidine and magrolimab/placebo, highlights the aggressive nature of the disease and the challenges in managing high-risk MDS patients.

Cytomegalovirus retinitis is a significant but relatively rare complication post-transplant, especially in non-HIV patients, despite the commonality of CMV infections among the general population [7]. The occurrence of CMV retinitis in this patient underscores the need for vigilant monitoring of CMV infections in post-transplant patients. The management of CMV retinitis in this patient, involving intravitreal injections, systemic antiviral therapy, and periodic fundus exams of both eyes to monitor for contralateral involvement, reflects the current standard of care. Past research has indicated that the treatment of CMV retinitis in immunocompromised patients typically involves a combination of systemic antiviral therapy and local ophthalmic treatments [11]. The use of foscarnet and maribavir in this case aligns with recommended practices, although the development of kidney toxicity highlights the potential complications associated with these treatments [12].

A case report by Vassallo et al. describes a similar scenario where a patient developed CMV retinitis post HSCT, necessitating a combination of systemic and intravitreal foscarnet and CMV-specific immunoglobulins (CMV-IVIG) [13]. This therapeutic approach achieved a significant improvement in visual acuity with no reported side effects, emphasizing the importance of combining systemic and local antiviral therapies for optimal outcomes. In comparison, our patient experienced significant kidney toxicity during foscarnet treatment, managed with hydration boluses. Although CMV-IVIG has been shown to be efficacious as a prophylaxis against cytomegalovirus reactivation in allogeneic HSCT patients and treatment of CMV viremia, its role in the treatment of active CMV retinitis remains unclear, warranting further research [14, 15].

The presence of TP53 mutations in this patient is significant, as these mutations are associated with poor prognosis in MDS and AML [10]. The persistence of TP53 mutations despite treatment and their eventual clearance post-transplant is an intriguing aspect of this case, suggesting a complex interplay between the genetic landscape of the disease and the patient’s response to therapy. Future studies could evaluate whether earlier or more aggressive targeting of TP53 mutations might alter the clinical course in similar patients.

Additionally, the potential development of GvHD, as indicated by the esophagogastroduodenoscopy (EGD)/colonoscopy findings, adds another layer of complexity to this case. Graft versus host disease is a common complication post transplant, and its management is critical for patient outcomes [16]. The use of steroids in this patient is in line with standard GvHD treatment protocols. However, it is notable that the prolonged use of immunosuppressive therapies may further compromise immune recovery and necessitate more vigilant CMV surveillance. 

## Conclusions

In summary, this case report contributes to the existing literature by highlighting the challenges in managing a patient with high-risk MDS transitioning to AML, complicated by post-transplant CMV retinitis and GvHD. It underscores the need for comprehensive management strategies that address both the hematological malignancy and the potential complications arising from treatment and post-transplant immunosuppression. Future research should focus on optimizing antiviral therapies for CMV retinitis, particularly in cases complicated by drug resistance, and evaluating adjunctive treatments, such as CMV-IVIG, to improve outcomes in this population. Additionally, standardizing protocols for early ophthalmologic screening in high-risk patients and incorporating routine monitoring for UL97 resistance in CMV-positive patients could aid in early detection and intervention clinical outcomes. These insights from this case provide valuable directions for enhancing the management of similar patients in clinical practice.
